# Two new and one little-known damsel bug of the subfamily Prostemmatinae Reuter (Hemiptera, Heteroptera, Nabidae) from China

**DOI:** 10.3897/zookeys.845.32893

**Published:** 2019-05-15

**Authors:** Ping Zhao, Runqian Mao, Liangming Cao

**Affiliations:** 1 Guangdong Key Laboratory of Animal Conservation and Resource Utilization, Guangdong Public Laboratory of Wild Animal Conservation and Utilization, Guangdong Institute of Applied Biological Resources, Guangzhou 510260, China Guangdong Institute of Applied Biological Resources Guangzhou China; 2 The Key Laboratory of Forest Protection, State Forestry and Grassland Administration of China, Research Institute of Forest Ecology, Environment and Protection, Chinese Academy of Forestry, Beijing 100091, China Kaili University Kaili China; 3 Department of Plant Protection, Kaili University, Kaili, Guizhou 556000, China Research Institute of Forest Ecology, Environment and Protection, Chinese Academy of Forestry Beijing China

**Keywords:** China, Nabidae, new species, Prostemmatinae

## Abstract

Two damsel bugs belonging to two genera of the subfamily Prostemmatinae from China are reported as new to science: Alloeorhynchus (Alloeorhynchus) yunnanensis**sp. n.** and *Rhamphocorisguizhouensis***sp. n.** The little-known species Alloeorhynchus (Alloeorhynchus) reinhardi Kerzhner & Günther, 1999 is redescribed. All species are illustrated in detail. Keys to the Chinese species of *Rhamphocoris* and *Alloeorhynchus* are provided aid in identification.

## Introduction

Prostemmatinae is a small subfamily in the family Nabidae with five genera and approximately 150 species worldwide (Schuh and Štys 1991; Kerzhner and Konstantinov 2008; Cassis 2016; Brailovsky and Barrera 2017); four genera and 27 species are found in the Palaearctic Region (Kerzhner 1996). However, only 17 species and four genera of the subfamily were reported in China prior to this study, mainly from Yunnan Province and adjacent southern provinces (Hsiao and Ren 1981; Ren 1998; Li et al. 2012; Kerzhner and Günther 1999). During fieldwork to Guizhou and Yunnan provinces, we collected several rare specimens of Prostemmatinae. Herein two new species are described, illustrated, and keyed. Alloeorhynchus (Alloeorhynchus) reinhardi Kerzhner & Günther, 1999 was described based on two short-winged females from Sichuan Province of China. We found a third macropterous female from Guizhou Province of China, which is redecribed herein.

Species of Prostemmatinae are considered primarily ground-inhabiting predators (Kerzhner 1996; Kerzhner and Konstantinov 2008). However, we found several specimens of *Rhamphocorisguizhouensis* under the bark of dead broadleaved evergreen trees along with an unknown species of Aradidae (Figs 19, 20), but nothing else is known about the biology of these three species.

## Materials and methods

The material examined in this study is now deposited in Guangdong Institute of Applied Biological Resources, Guangzhou, China. The external structures were examined using a binocular dissecting microscope. Male genitalia were soaked in hot 90% lactic acid for approximately ten minutes to remove soft tissue, then in hot distilled water, and dissected under a microscope. The dissected parts of the genital structures were placed in a plastic microvial with lactic acid under the corresponding specimen. All drawings were traced with the aid of a camera lucida. Measurements were obtained using a calibrated micrometer. Body length was measured from the apex of head to the tip of the hemelytra in resting position. Maximum width of the pronotum was measured across humeral angles. All measurements are given in millimeters. Classification system and morphological terminology mainly follow those of Ren (1998) and Kerzhner (1992). The tribal, generic, and specific names in the text are arranged alphabetically.

## Taxonomy

### Subfamily Prostemmatinae Reuter, 1890

#### Tribe Phorticini Kerzhner, 1971

##### Genus Rhamphocoris

Taxon classificationAnimaliaPasseriformesAlaudidae

Kirkaldy, 1901

Rhamphocoris Kirkaldy, 1901: 221; Ren 1998: 47; Cassis 2016: 172. Type species: Rhamphocorisdorothea Kirkaldy, 1901, by monotypy.
Aristonabis Reuter & Poppius, 1909: 48 (syn. by Kerzhner 1970: 280). Type species: Aristonabispulcher Reuter & Poppius, 1909, by original designation.
Harrisiella China & Miller, 1953: 115 (syn. by Kerzhner 1970: 280). Type species: Harrisiellahumeralis China & Miller, 1953, by original designation.

###### Diagnostic characters.

**Figures 1–4.**
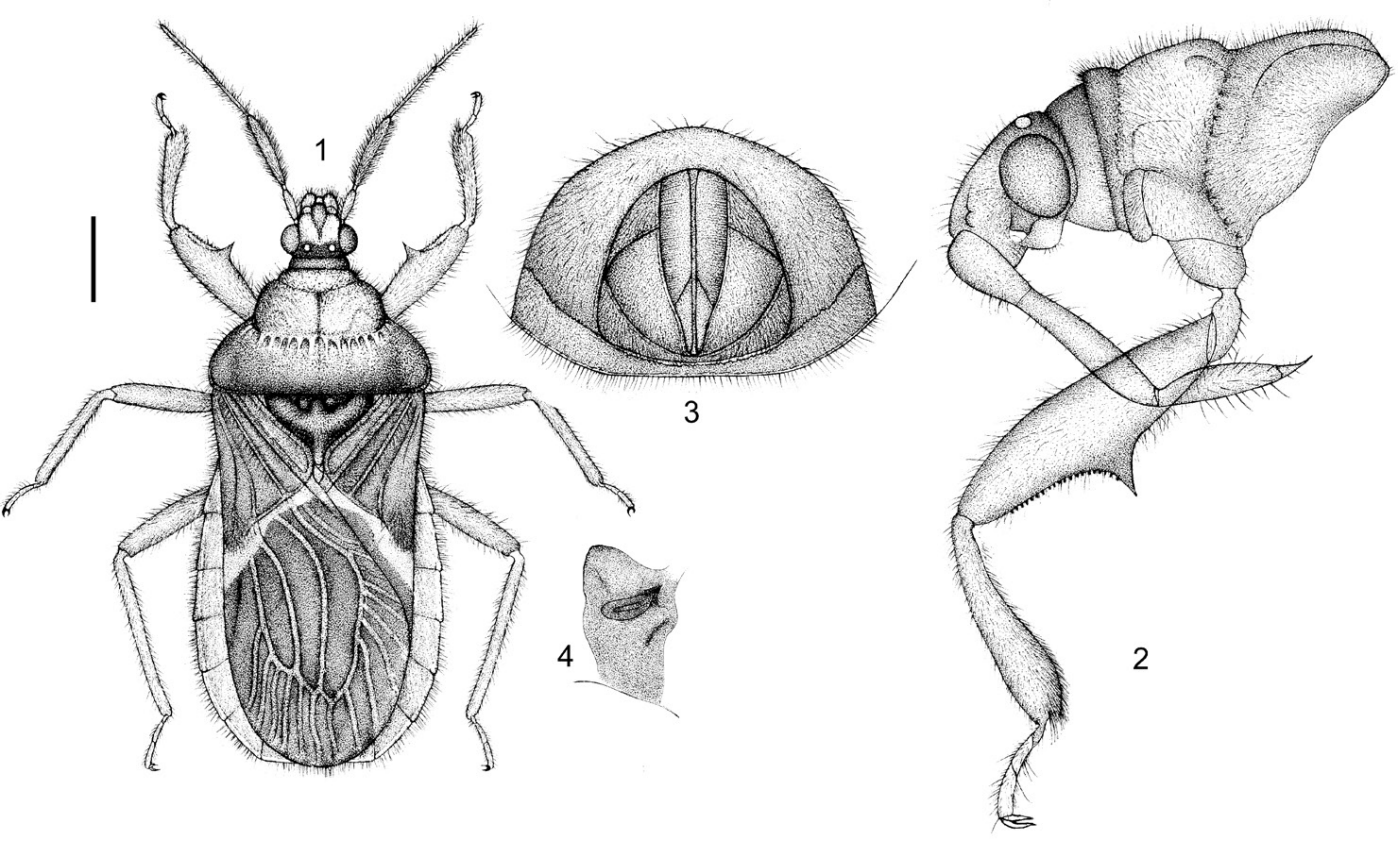
*Rhamphocorisguizhouensis* sp. n., female holotype. **1** habitus, dorsal view **2** head and pronotum, antennae removed, lateral view **3** apical part of abdomen, ventral view **4** ostiolar peritreme of metathoracic scent gland. Scale bars: 0.87 mm (**1**); 0.48 mm (**2**); 0.40 mm (**3, 4**).

Body elongate-oblong, flattened dorso-ventrally; body shiny, black or red with yellow markings (Figs 1, 19). Head slightly declined anteriorly (Fig.&nbsp;2); rostrum 4-segmented, first segment short and thickened, second segment longest, third approximately half length of second, fourth segment shortest (Fig. 2); first and second antennal segments distinctly thickened and thicker than third or fourth segments (Fig. 1). Pronotum constricted slightly between collar and anterior pronotal lobe and distinctly between anterior lobe and posterior lobe (Figs 1, 2); posterior pronotal lobe distinctly wider than anterior lobe; anterior lobe with middle longitudinal sulcus, sides slightly anteriorly bulged; posterior lobe laterally roundly produced, posterior margin straight; scutellum subtriangular, sub-basally with two small round depressions, apical part strongly acute. Hemelytra oval, membrane with three elongate cells (Fig. 1); fore femur beneath with a large acute angular process medially and with two lines of small dentate tubercles from median angular process to apex of femur; fore tibiae slightly curved, apex dilated, without spongy fossula; ostiolar peritreme gradually posteriorly widened, elongated, somewhat curved (Fig. 4).

###### Remarks.

*Rhamphocoris* is a small genus of the subfamily Prostemmatinae with ten known species worldwide. Five species (including the new species described herein) have been recorded in China, *R.borneensis* (Schumacher, 1914) [China (Yunnan, Hainan); Malaysia, Vietnam], *R.elegantulus* (Schumacher, 1914) [China (Taiwan)], *R.hasegawai* (Ishihara, 1943) [China (Yunnan, Taiwan)], *R.tibialis* Hsiao, 1981 [China (Yunnan)], and *R.guizhouensis* sp. n. [China (Guizhou)] (Hsiao and Ren 1981; Ren 1998).

##### Key to the Chinese Species of *Rhamphocoris* Kirkaldy

**Table:** 

1	Pronotum bicolored, black with anterior pronotal lobe and basal part of posterior lobe red	***R.guizhouensis* sp. n.**
–	Pronotum unicolorous, uniformly red or black	**2**
2	Pronotum black	***R.hasegawai* (Ishihara, 1943)**
–	Pronotum red	**3**
3	Tibiae blackish brown	***R.tibialis* Hsiao, 1981**
–	Tibiae red	**4**
4	Scutellum red	***R.borneensis* (Schumacher, 1914)**
–	Scutellum black	***R.elegantulus* (Schumacher, 1914)**

###### Rhamphocoris guizhouensis sp. n.

Taxon classificationAnimaliaPasseriformesAlaudidae

http://zoobank.org/CC9D5DE7-61D7-420C-91F4-D30A3E158A6D

Figs 1–4 , 19 , 20

####### Type material.

**Holotype**: female, China, Guizhou, Liping, Taiping Mountain, 28-VII-2009, 26°14'19.54"N, 109°18'38.59"E, Zhao Ping leg.

####### Diagnosis.

Body length 5.48 mm. Body color red with black markings, shiny; head and pronotum bicolored, mostly red, but vertex of head, dorsal surface of neck, anteocular area of head, collar, most of posterior pronotal lobe blackish brown to black; scutellum black.

####### Description.

**Color.** Body red, shiny. First antennal segment pale yellow, second antennal segments blackish brown, third to fourth antennal segments pale brown; vertex of head, dorsal surface of neck, anteocular area, eyes, anterior margin of collar of pronotum, propleural epimeron, pleuron and sternum of meso- and metathorax, fore wing (except strip or markings on basal part of membrane and basal part of clavus yellowish white) dark brown to blackish brown (Figs 1, 19); posterior pronotal lobe (except basal part), anterior margin of each abdominal segment pale brown to brown; scutellum, small spines of underside of fore femur blackish brown to black; abdomen ventrally pale yellowish brown and laterally blackish brown; strip of basal part of membrane yellowish white (Figs 1, 19), basal part of clavus pale yellowish brown.

**Structure.** Body clothed with golden yellow setae. Body flattened dorsoventrally. Head with rounded processes ventrally (Fig. 2); pronotum distinctly transversely constricted between collar and anterior lobe and between anterior and posterior lobe; anterior pronotal lobe bulged, arcuately laterally shallowly sulcate, medially longitudinally sulcate; scutellum sub-basally concave and with two small rounded depressions, apical part produced; fore femur somewhat thickened, and beneath with acute angular process. Abdomen oblong, not covered completely by fore wing; posterior margin of abdomen in female straight; fore wing reaches to abdominal tip. Ostiolar peritreme of metathoracic scent gland shown in Fig. 4.

**Measurements** Female, n = 1. Body length 5.48; maximal width of abdomen 3.33. Length of head 0.71; width of head 0.71; length of anteocular part 0.31; length of postocular part 0.05; length of neck 0.16; length of synthlipsis 0.38; interocellar space 0.19; length of antennal segments I–IV= 0.33, 0.76, 0.67, 0.55; length of rostral segments I–IV=0.31, 0.71, 0.48, 0.19; length of collar 0.19; length of anterior lobe of pronotum 0.48; length of posterior lobe of pronotum 0.67; maximal width of thorax 2.19; length of scutellum 0.67; length of hemelytron 3.76.

####### Male.

Unknown.

####### Distribution.

China (Guizhou).

####### Etymology.

The specific name is derived from the type locality of the species.

####### Remarks.

The new species resemble to *R.hasegawai* in body shape and color, but in the latter the head and pronotum are totally black. The new species is similar to *R.elegantulus* but easily distinguished by the body color: the head (except the vertex) is black; the collar and most of the posterior lobe of pronotum black, the anterior lobe and basal part of posterior lobe red (Fig. 19) (vs. the head and pronotum totally red in *R.elegantulus*) (Ren 1998). The five Chinese species in the genus *Rhamphocoris*, including the new one described herein, can be distinguished in the above key. The new species is flattened and the single specimen was collected together with the flat bug *Aradus* sp. (Aradidae) under the bark, and possibly feeds on flat bugs. The fifth-instar nymph is red except for the brown to yellow antennae and the blackish brown wing pads (Fig. 20).

#### Tribe Prostemmatini Reuter, 1890

##### Genus Alloeorhynchus

Taxon classificationAnimaliaPasseriformesAlaudidae

Fieber, 1860

Alloeorhynchus Fieber, 1860: 43; 1861: 159; Ren 1998: 51; Distant 1904. Type species: Piratesflavipes Fieber, 1836, by subsequent monotypy (Fieber 1861: 159).
Falda Gross, 1954: 139 (syn. by Kerzhner 1970: 282). Type species: Faldaqueenslandica Gross, 1954, by original designation.

###### Diagnostic characters.

Body elongate oblong. Anterior part of head strongly declined, or somewhat declined; anteocular area of head short, nearly conical; posterior margin of eyes adjacent to anterior margin of pronotum; ocelli present; antennae clothed with long setae, first antennal segment short, extending beyond apex of head; rostrum slender, extending to metasternum, first segment short and thick, second and third segments longest, fourth segment short; pronotum distinctly constricted transversally behind middle, posterior margin straight; scutellum long, subequal to width at base. Fore and mid femora moderately thickened, underneath with two to three rows of small spines; fore tibia slightly shorter than femur, apical part widened, with spongy fossula.

###### Remarks.

The genus includes two subgenera, *Alloeorhynchus* and *Psilistus*, and 49 species in the world (Ren 1998; Brailovsky and Barrera 2017). Eight species have been recorded in China, including one new and one little-known species described in the present study: A. (A.) notatus Distant, 1919 [China (Yunnan); India, Nepal], A. (A.) sinicus Ren, 1998 [China (Zhejiang)], A. (A.) vinulus Stål, 1864 [China (Hainan, Taiwan); Japan, Vietnam, Java, Philippines, Burma], A. (A.) yunnanensis sp. n. [China (Yunnan)], A. (A.) reinhardi Kerzhner & Günther, 1999, and A. (P.) corallinus (Stål, 1873) [China (Yunnan); Burma, Sikkim, India, Malaysia], Alloeorhynchus (P.) bakeri Harris 1930 [China (Yunnan)] (Hsiao et al. 1981; Ren 1998; Kerzhner and Günther 1999; Gapon and Konstantinov 2008; Li et al. 2012).

##### Key to the Chinese Species of *Alloeorhynchus* Fieber

**Table:** 

1	Middle of fore and mid femora ventrally not dentate or without horn-like extensions	**2**
–	Middle of fore and mid femora ventrally dentate or with horn-like extensions	**3**
2	Body red, transverse constriction on pronotum distinct, apex of scutellum with two small tubercles	**Alloeorhynchus (P.) corallinus (Stål, 1873)**
–	Body black, transverse constriction on pronotum indistinct, apex of scutellum round, without tubercles	**Alloeorhynchus (P.) bakeri Harris, 1930**
3	Head blackish brown to black	**4**
–	Head yellow	**6**
4	Anterior pronotal lobe yellow or pale yellowish brown	***A.*** (***A.***) ***vinulus* Stål, 1864**
–	Anterior pronotal lobe blackish brown	**5**
5	Posterior pronotal lobe blackish brown, middle part paler; fore wing brown, middle part paler	**A. (A.) sinicus Ren, 1998**
–	Posterior pronotal lobe blackish brown to black; fore wing blackish brown, basal part paler	**A. (A.) reinhardi Kerzhner & Günther, 1999**
6	Basal part of corium distinctly yellow; fore femur beneath with three distinct tubercles	**A. (A.) notatus Distant, 1919**
–	Basal, middle and apical part of corium with obscure yellow markings; fore femur beneath with four distinct tubercles	**A. (A.) yunnanensis sp. n.**

###### Alloeorhynchus (Alloeorhynchus) reinhardi

Taxon classificationAnimaliaPasseriformesAlaudidae

Kerzhner & Günther, 1999

Figs 5 , 6–9 , 21

Alloeorhynchus (Alloeorhynchus) reinhardi Kerzhner & Günther, 1999, 33: 221; Gapon and Konstantinov 2008: 24.

####### Material examined.

1 female, China, Guizhou, Kaili, 3-III-2011, 26°34'15.93"N, 107°58'34.53"E, Zhao Ping leg.

####### Diagnosis.

Body blackish brown with pale yellowish-brown markings; head, thorax, scutellum, fore wing (except basal part of corium), and lateral sides of abdominal sterna blackish brown to black; antennae, rostrum, and legs yellow.

####### Redescription.

**Figure 5.**
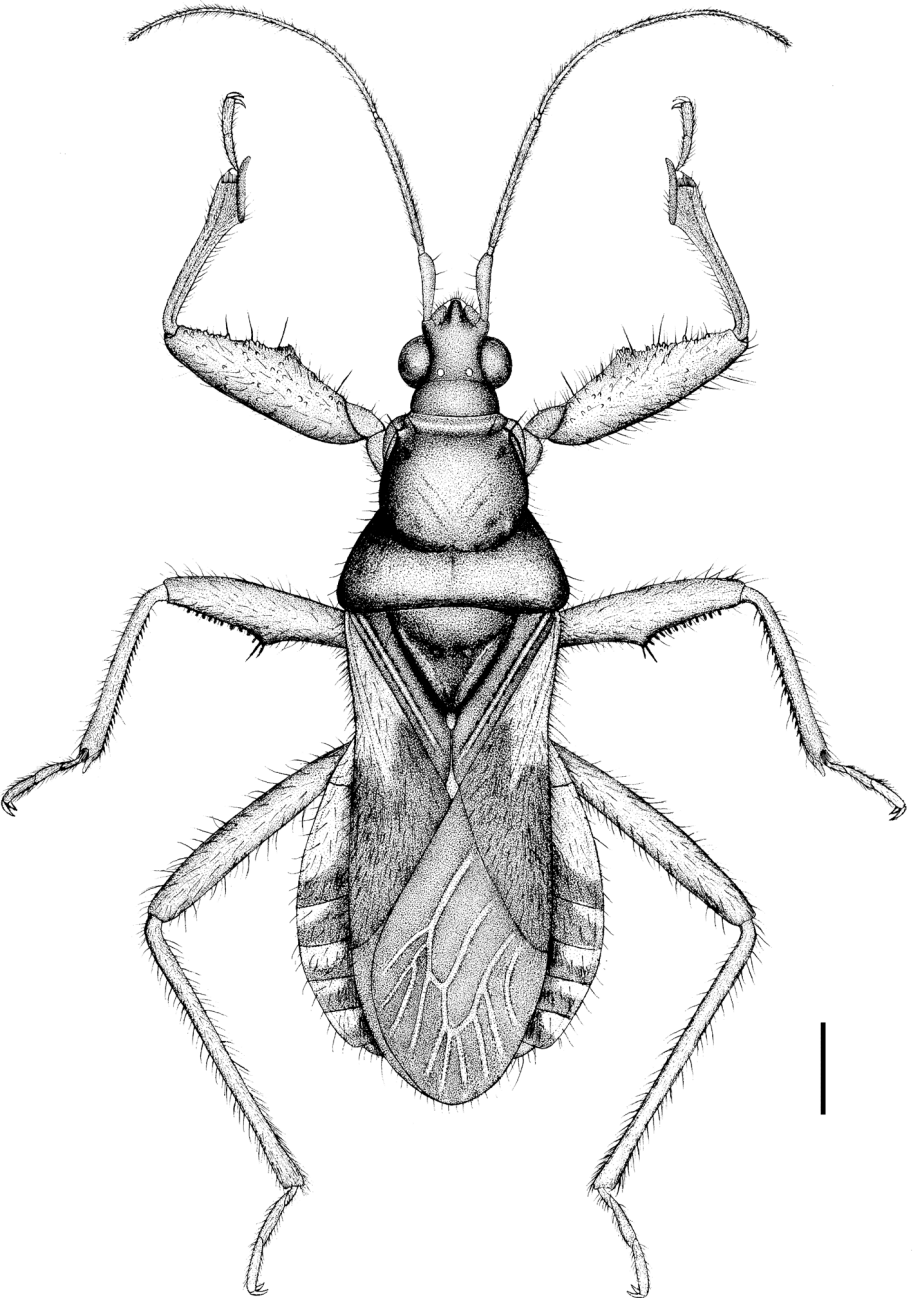
Alloeorhynchus (Alloeorhynchus) reinhardi Kerzhner & Günther, 1999, female, habitus, dorsal view. Scale bar: 0.67 mm.

**Color.** Body blackish brown to black (Figs 5, 21). Head, first rostral segment, thorax, scutellum, corium (except basal part), clavus, membrane, spines beneath femora and tibia, apical part and sides of abdominal sterna (Fig. 7), apical part of third to seventh connexival segments, eighth connexival segment blackish brown to black (Figs 5, 7, 21); second to fourth antennal segments, apical part of femora, apical and basal parts of tibiae, tarsi pale yellowish brown; first antennal segment, second to fourth rostral segments, coxae, trochanters, femora (except apical part), tibiae (except basal and apical parts), basal part of corium, middle part of abdominal sternum (third to sixth segments), second connexival segment, basal part of third to seventh connexival segments yellow.

**Figures 6–9.**
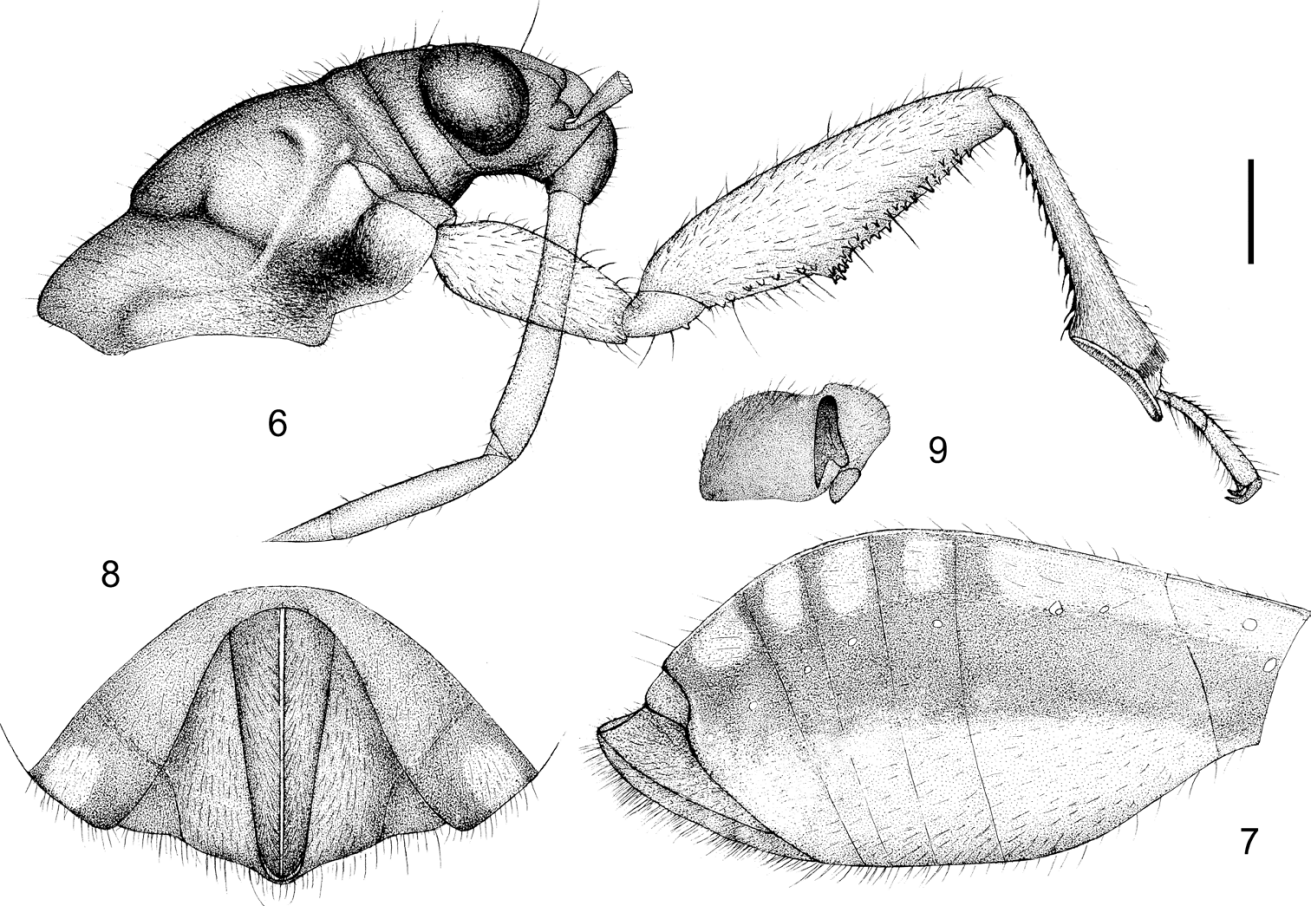
Alloeorhynchus (Alloeorhynchus) reinhardi Kerzhner & Günther, 1999, female. **6** head and pronotum, antennae removed, lateral view **7** abdomen, lateral view **8** apical part of abdomen, ventral view **9**&nbsp;Ostiolar peritreme of metathoracic scent gland. Scale bar: 0.32 mm (**6**); 0.8 mm (**7**); 0.4 mm (**8**); 0.32 mm (**9**).

**Structure and vestiture.** Macropterous. Body elongate oblong, posteriorly gradually widened (Fig. 5). Head, pronotum, ventral surface of abdomen, legs and antennae smooth and shiny (Fig. 21). Body sparsely clothed with white long setae; first antennal segment sparsely clothed with oblique setae, second to fourth segments densely clothed with oblique setae; tibiae and corium of fore wing clothed densely with setae (Fig. 5). Subapical part of first antennal segment curved outward. Anterior pronotal lobe somewhat bulged and twice as long as posterior pronotal lobe; scutellum sub-angular, apical part with small protuberance. Fore coxa strong, subequal to 2/5 of femur in length; fore and mid femora distinctly thickened, ventrally sub-basal 2/5 dilated in a protrusion, and apical half beneath with two lines of black dentate spines; fore tibia apically dilated with spongy fossula and underneath with two lines of black spines; mid tibia beneath with a line of distinct spines and a line of indistinct spines (Fig. 6). Abdomen in female widened posteriorly; fore wing extending to tip of abdomen (Figs&nbsp;5, 7). Ostiolar peritreme of metathoracic scent gland shown in Fig. 9. Apical part of abdomen in female shown in Figs 7, 8.

**Measurements.** Female, n = 1. Body length 5.70; maximal width of abdomen 2.30. Length of head 0.93; length of anteocular part 0.83; length of postocular part 0.50; length of synthlipsis 0.40; interocellar space 0.15; length of antennal segments I–IV= 0.60, 1.07, 0.97, 1.40; length of rostral segments I–IV=0.33, 1.07, 0.76, 0.27; length of collar 0.20; length of anterior lobe of pronotum 0.90; length of posterior lobe of pronotum 0.50; maximal width of thorax 1.73; length of scutellum 0.87; length of hemelytron 3.93.

####### Distribution.

China (Guizhou, Sichuan).

####### Remarks.

Kerzhner and Günther (1999) described the species A. (A.) reinhardi in German based on two short-winged females collected from Sichuan Province in Southern China. We found a macropterous female in Guizhou Province, which is redescribed here in English to facilitate identification. We identified this species by comparing it with the description and the color illustration in the paper published by Kerzhner and Günther (1999) with the help of Dr Steffen Roth (University Museum of Bergen), but we were unable to examine the type specimens.

###### Alloeorhynchus (Alloeorhynchus) yunnanensis sp. n.

Taxon classificationAnimaliaPasseriformesAlaudidae

http://zoobank.org/D668063A-B4A4-4AEF-AA89-F18CD5BD3834

Figs 10 , 11–18

####### Type material.

**Holotype**, male, China, Yunnan, Xishuangbannan, Mengla, Mengman Town, Nanping Village, 23-IV-2013, 21°17'18.86"N, 101°17'48.86"E, Wan Renjing and Zhao Ping leg.

####### Diagnosis.

Corium reddish brown, and its basal, middle and apical part with obscure yellow markings; fore femur beneath with four distinct tubercles; head greyish yellow, anterior pronotal lobe greyish brown with median longitudinal part yellow.

####### Description.

**Figure 10.**
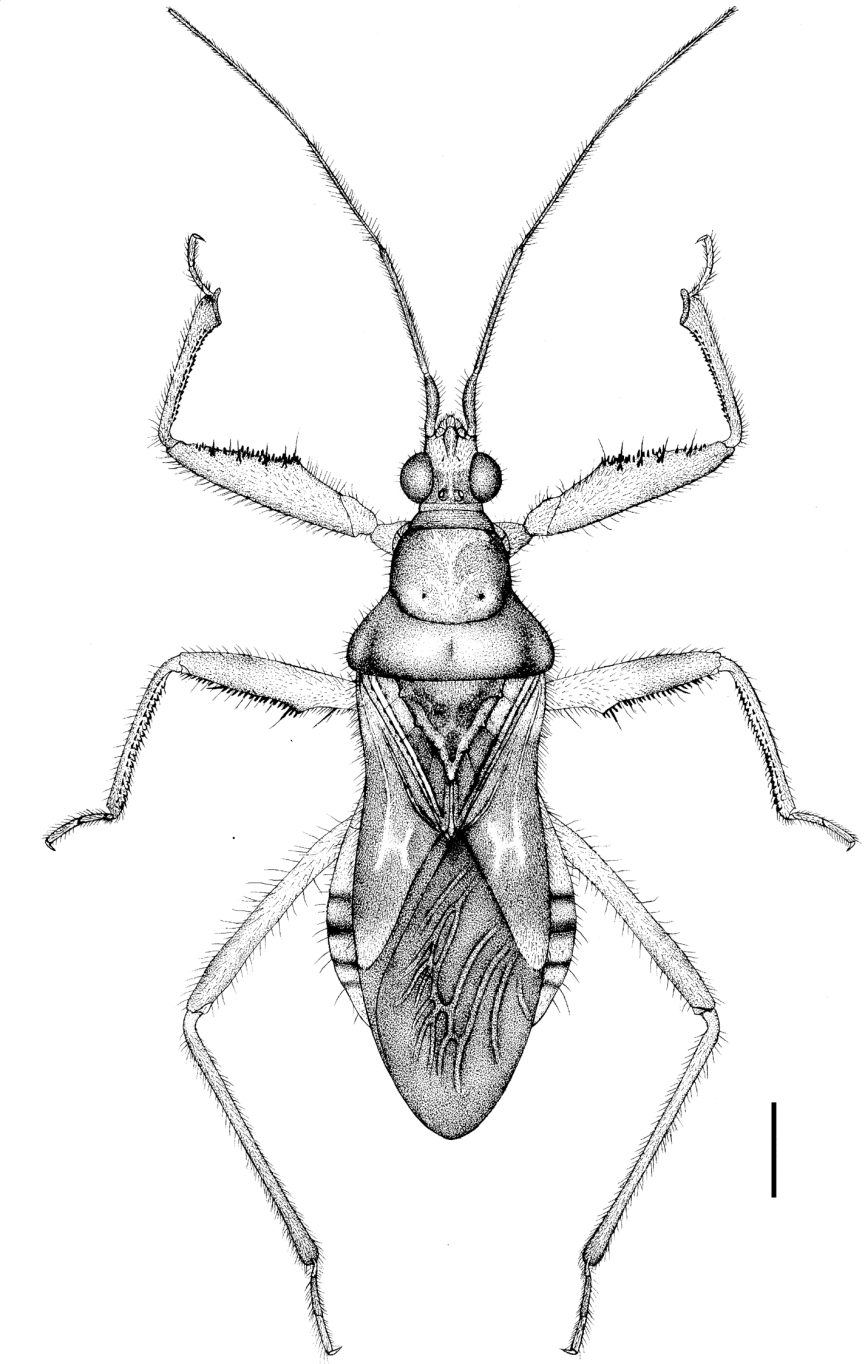
Alloeorhynchus (Alloeorhynchus) yunnanensis sp. n., male holotype, habitus, dorsal view. Scale bar: 0.8 mm.

**Figures 11–18.**
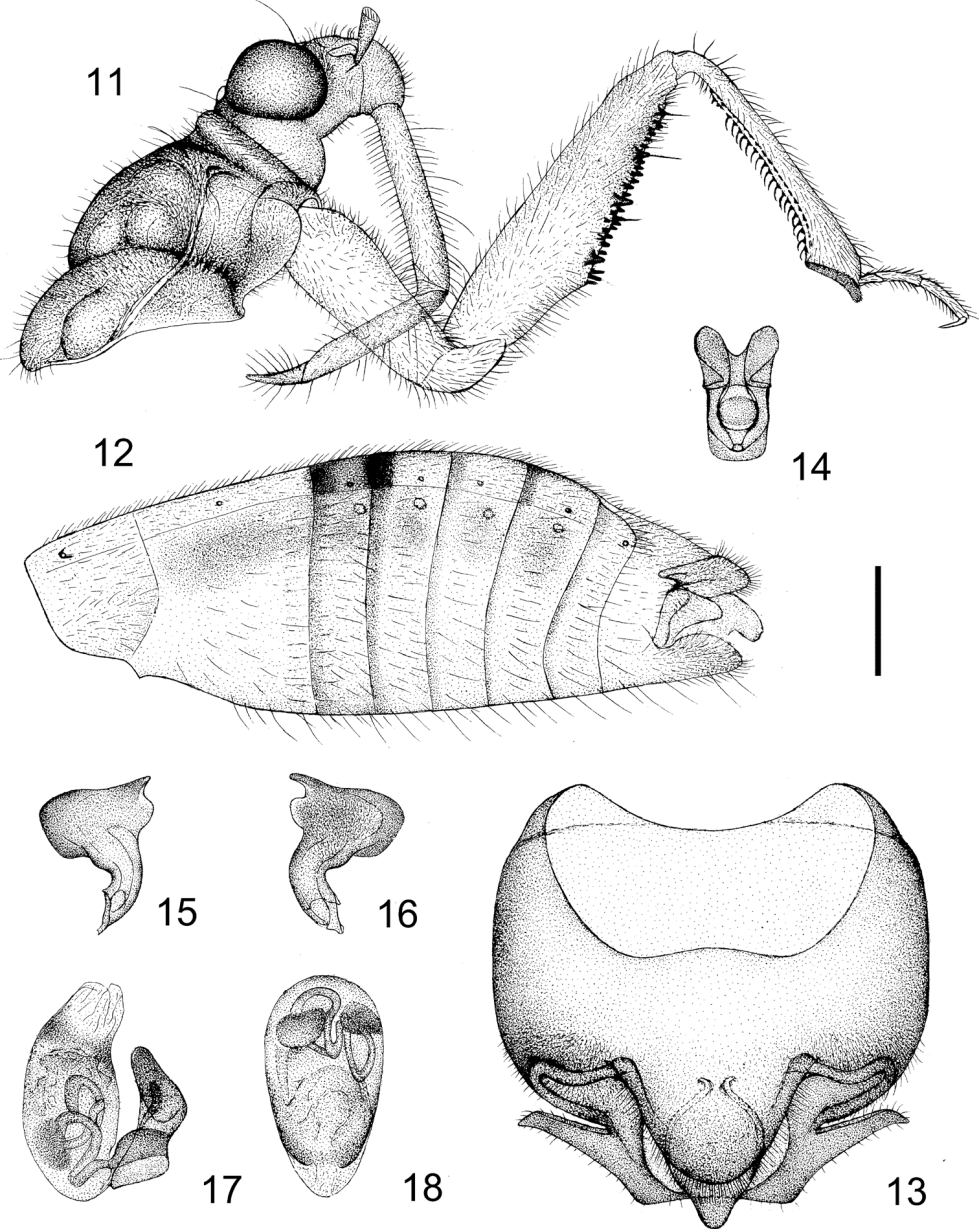
Alloeorhynchus (Alloeorhynchus) yunnanensis sp. n., male holotype. **11** head and pronotum, antennae removed, lateral view **12** abdomen, lateral view **13** pygophore, ventral view **14** phallobase **15, 16** paramere **17** phallus, lateral view **18** phallus, ventral view. Scale bar: 0.50 mm (**11**); 0.40&nbsp;mm&nbsp;(**12**); 0.26 mm (**13–18**).

**Color.** Body greyish brown dorsally and pale yellowish ventrally. Basal, middle, and apical markings of corium, apical part of femur, basal part of tibia, two sides of abdomen ventrally tinged with red (Figs 10, 12). Antennae, neck dorsally, thorax (except metapleuron somber black), scutellum, corium (except markings), clavus (except basal part), subapical part of femur, hind tibia (except basal part), hind tarsus, basal part of fourth to seventh connexival segments brown (Figs 10, 12); spines beneath femur, tibiae of fore and mid legs, membrane black (Figs 10–12); head (except neck dorsally), rostrum, coxae, trochanters, femora (except apical part), fore and mid tibiae, markings of corium, middle part of abdominal sternum, second connexival segment, apical part of third to seventh connexival segments yellow.

**Structure and vestiture.** Body elongate oblong (Fig. 10). Body clothed with yellowish to white setae; first antennal segment sparsely clothed with oblique setae, second to fourth segments densely clothed with oblique setae; scutellum and corium of fore wing clothed with blackish setae (Figs 10–12). Subapical part of first antennal segment somewhat curving outward. Pronotum smooth and shiny, anterior pronotal lobe somewhat bulged; scutellum sub-angular, apical part somewhat produced posteriorly. Fore coxa strong, subequal to 1/2 of femur in length; fore and mid femora thickened and ventrally sub-basal 2/5 dilated in a protrusion, and fore femur beneath inside with four small short spines and outside with numerous black denticles from the protrusion to apical part of femur; fore tibiae apically dilated with spongy fossula and beneath with two lines of black spines; mid tibiae beneath with a line of distinct spines and a line of indistinct spines (Figs 10, 11); fore wing extending beyond tip of abdomen. Pygophore round, median pygophore process broad and produced acutely laterally (Fig.&nbsp;13); paramere triangulate, apical part dilated (Figs 17, 18); basal plate of phallobase short and thick, pedicel short (Figs 14, 17). Phallosome elliptic, shown in Figs 17, 18.

**Measurements.** Male, n = 1. Body length 5.86; maximal width of abdomen 2.00. Length of head 0.80; length of anteocular part 0.27; length of postocular part 0.20; length of synthlipsis 0.33; interocellar space 0.07; length of antennal segments I–IV= 0.53, 1.20, 1.13, 1.71; length of rostral segments I–IV=0.33, 0.97, 0.73, 0.27; length of anterior lobe of pronotum 0.93; length of posterior lobe of pronotum 0.53; maximal width of thorax 1.73; length of scutellum 1.00; length of hemelytron 4.00.

**Female.** Unknown.

####### Distribution.

China (Yunnan).

####### Etymology.

The specific name refers to the type locality of the new species.

####### Remark.

The general body shape and the structure of fore leg resemble those of Alloeorhynchus (Alloeorhynchus) maculosus Kerzhner, 1992 (India, Sumatra). In the new species the neck of the head is greyish, the anterior pronotal lobe is greyish brown with pale markings, the markings on the corium of fore wing is obscure, and the fourth to seventh connexival segments are yellow basally with brown markings (vs. the anterior pronotal lobe yellowish and its anterior margin darker, the head is yellow, the markings on the corium of fore wing are distinct, and the fourth to fifth connexival segments have brown markings in A. (A.) maculosus). The species is also similar to Alloeorhynchus (Alloeorhynchus) fuscescens Kerzhner, 1992 (Nepal, Vietnam) and Alloeorhynchus (Alloeorhynchus) himalayensis Kerzhner, 1992 (northern India) in the body coloration and the body size, but the paramere in the new species is triangular with an apical protuberance (Figs 15, 16) [vs. the paramere clavate without an apical protuberance in A. (A.) fuscescens and A. (A.) himalayensis]. The fore femur of the new species is with many small spines and four distinct larger spines [vs. the fore femur beneath with many small spines but without four distinct larger spines in A. (A.) distanti Harris, 1940 (Northern India and Nepal)] (Kerzhner 1992).

**Figures 19–21.**
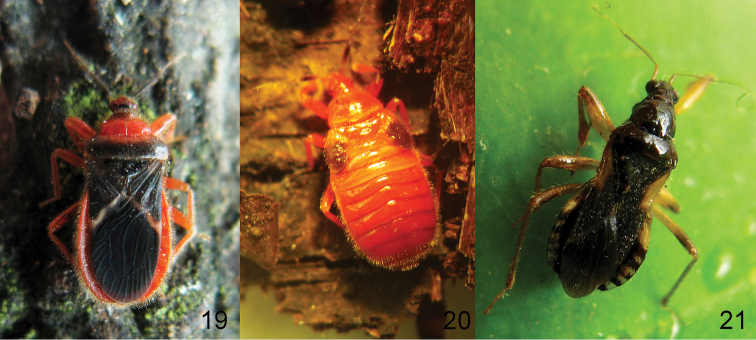
**19**, **20**, *Rhamphocorisguizhouensis* sp. n., female holotype; **21**Alloeorhynchus (Alloeorhynchus) reinhardi Kerzhner & Günther, 1999, female **19, 21** adult **20** fifth-instar nymph.

## Supplementary Material

XML Treatment for Rhamphocoris

XML Treatment for Rhamphocoris guizhouensis

XML Treatment for Alloeorhynchus

XML Treatment for Alloeorhynchus (Alloeorhynchus) reinhardi

XML Treatment for Alloeorhynchus (Alloeorhynchus) yunnanensis
